# Combination of 0.05% Azelastine and 0.1% Tacrolimus Eye Drops in Children With Vernal Keratoconjunctivitis: A Prospective Study

**DOI:** 10.3389/fmed.2021.650083

**Published:** 2021-09-17

**Authors:** Minjie Chen, Anji Wei, Bilian Ke, Jun Zou, Lan Gong, Yan Wang, Chaoran Zhang, Jianjiang Xu, Jia Yin, Jiaxu Hong

**Affiliations:** ^1^Department of Ophthalmology, Eye and Ear, Nose, and Throat Hospital of Fudan University, Shanghai, China; ^2^Key Laboratory of Visual Impairment and Restoration of Shanghai, Fudan University, Shanghai, China; ^3^Key myopia Laboratory of NHC, Shanghai, China; ^4^Key Laboratory of Myopia, Chinese Academy of Medical Science, Shanghai, China; ^5^Department of Ophthalmology, Shanghai General Hospital, Shanghai Jiaotong University School of Medicine, Shanghai, China; ^6^Department of Ophthalmology, Shanghai Tenth People's Hospital, Tongji University School of Medicine, Shanghai, China; ^7^Massachusetts Eye and Ear, Harvard Medical School, Boston, MA, United States; ^8^Department of Ophthalmology, The Affiliated Hospital of Guizhou Medical University, Guizhou, China

**Keywords:** vernal conjunctivitis, tacrolimus, azelastine, ocular surface disease index, palpebral conjunctival papillae

## Abstract

**Aims:** To compare the efficacy of the combination of 0. 05% azelastine and 0.1% tacrolimus eye drops with 0.1% tacrolimus monotherapy in pediatric patients with vernal keratoconjunctivitis (VKC).

**Methods:** Prospective study. Seventy-six patients with VKC were randomized 1:1 into monotherapy group with 0.1% tacrolimus or combination therapy group with 0.1% tacrolimus and 0.05% azelastine. The Ocular Surface Disease Index (OSDI) scores and the signs of conjunctival hyperemia, corneal involvement, and palpebral conjunctiva papillae were assessed at baseline and at 1, 2, and 6 weeks after treatment.

**Results:** Two groups were comparable in age, sex, duration of VKC, OSDI, and clinical signs of VKC at baseline. Significant improvements in OSDI score and clinical signs were observed in both groups at all follow-up visits (all *p* < 0.001), compared with baseline. The combination therapy group showed a larger decrease in OSDI score from baseline (10.30 ± 0.9) compared with monotherapy group (7.30 ± 0.7, *p* =0.0085) at 1 week. Greater improvements in conjunctival hyperemia and conjunctival papillae were identified in the combination therapy group, compared with in the monotherapy group, at all follow-up visits (all *p* < 0.05). The corneal involvement scores in the combination group is significantly lower than the monotherapy group at 2 weeks after the treatment (*p* = 0.0488). No severe adverse effect was found in either group during the study.

**Conclusions:** Compared with a monotherapy of 0.1% tacrolimus, the combination of 0.05% azelastine and 0.1% tacrolimus eye drops lead to faster and greater improvements in clinical signs and symptoms of vernal keratoconjunctivitis in pediatric patients.

## Introduction

Allergic conjunctivitis affects about 10–20% of the population and is associated with poorer performance in school, difficulties in social communication, and high rates of anxiety and depression (1, 2). As one of the severe forms of allergic conjunctivitis, vernal keratoconjunctivitis (VKC) can have a prolonged course that is refractory to routine treatment strategies, leading to corneal shield ulcers, scarring, and even blindness (3).

So far, topical steroids are a main therapy for VKC, however, they do not always achieve a complete remission, especially for the most severe form with corneal involvement. In addition, topical steroids usually require months or years in treatment, leading to infectious episodes and elevations of intraocular pressure (IOP). More specifically, in the case of children and adolescents, the reported incidence of steroid-induced glaucoma is higher than that in adults, close to 2% (4, 5). Besides, steroid-induced cataract has also raised the level of concern (6).

Topical tacrolimus as an alternative to topical steroids has proven to be efficacious while not causing IOP rises (7–9). It is commercially available e.g., in Japan and China. Unfortunately, the tacrolimus formulation is known to cause irritation and epithelial keratitis, leading to low compliance in pediatric patients (10). Furthermore, it is well known that tacrolimus achieves immunosuppression mainly by inhibiting T lymphocyte activation due to inhibition of interleukin 2 transcription, which decreases T lymphocyte responsiveness to foreign antigens. Therefore, this process takes days and weeks to reach full therapeutic effect (11).

The purpose of this study was to evaluate the efficacy of topical 0.1% tacrolimus alone or with adjuvant 0.05% azelastine, a topical dual-acting antihistamine/mast-cell stabilizers, in children and adolescents with VKC. We hypothesize that the combination therapy may be more effective than tacrolimus monotherapy and lead to an earlier remission in patients' symptoms and signs. To accomplish this, we conducted a prospective study in which patients received topical tacrolimus with or without azelastine.

## Patients and Methods

### Study Design and Patients

This was a prospective study conducted in three centers in China. This study was registered at www.chictr.org.cn (clinical trial accession number: ChiCTR900022169) and was approved by the Institutional Review Board of the Eye & ENT Hospital of Fudan University. All procedures were performed in accordance with the Declaration of Helsinki and with the approved research protocol. For all children, at least one parent or legal guardian provided written informed consent, and patients provided written informed assent.

The study enrolled 76 patients (aged 5–17 years) with newly-onset vernal keratoconjunctivitis (VKC), who were visiting Eye and ENT Hospital, Shanghai General Hospital and Shanghai Tenth People's Hospital in eastern China from April 1, 2019 to October 30, 2019. Patients were randomized to receive either 0.1% tacrolimus (three times daily) or 0.05% azelastine (0.05% azelastine hydrochloride, MEDA Pharma GmbH & Co. KG) (twice daily) plus 0.1% tacrolimus (0.1% tacrolimus, Eye and ENT Hospital, Shanghai, China) (three times daily) in a 1:1 ratio according to a computer-generated predetermined randomization list. VKC was diagnosed based on the following criteria: symptoms of redness, itching, photophobia, tearing, foreign body sensation, burning, and signs of conjunctival hyperemia, palpebral conjunctiva papillae, Horner-Tranta spots, corneal involvement (including the presence of a shield ulcer, exfoliation superficial punctate keratitis and superficial keratitis).

Patients using systemic steroids or any immunosuppressive drugs or non-steroidal anti-inflammatory medications, or having associated corneal diseases, uveitis, glaucoma, and optic atrophy were excluded from the study. All the participating patients were diagnosed with active disease at the time of enrolment. Any prior topical medication for VKC was stopped for 3 days and only physical measures (were advised during that period.

### Outcome Measures

Subjects were assessed at baseline and after 1, 2, and 6 weeks of therapy. Both eyes of each patient were examined. All examinations were conducted by the experienced operators. Ocular signs (conjunctival hyperemia, corneal involvement and palpebral conjunctiva papillae) were measured using a 4-point scale at all visits (8). Two expert ophthalmologists independently attributed objective scores, and in case of disagreements a mean value was assigned. The Ocular Surface Disease Index (OSDI) was conducted at all visits. The questionnaire has three subscales: ocular symptoms, vision-related function, and environmental triggers. Patients rated their responses on a scale from 0 (“never”) to 4 (“all of the time”) assisted by the patients' guardian. A final score is calculated regarding the answers (11). All adverse events, either reported by participants or observed by researchers at each visit, were recorded. The relationship between the adverse events and the study medication was also assessed.

The primary outcomes were the changes in OSDI score from baseline at 1, 2, and 6 weeks after therapy. The secondary outcomes included improvements in conjunctival hyperemia, corneal involvement and palpebral conjunctival papillae at 1, 2, and 6 weeks after therapy.

### Statistical Analyses

Statistical analyses were performed using Stata 14.0 (Stata Corp., College Station, TX, USA). Quantitative data were expressed as the mean ± SD. The worst eye was defined as the study eye with the worst total signs values. The right eye was selected as the study eye if both eyes had the identical signs scores. Analysis of variance (ANOVA) or the Kruskal–Wallis test was used to test for difference among four different follow-ups, and the Bonferroni test was used to identify which pairs were significantly different. Paired *t* test or the matched-pairs signed-rank test was used to identify between-group differences. Numeration data were compared between the two groups using χ2 tests. Statistical significance was assumed at *p* < 0.05.

## Results

### Baseline Characteristics

**Figure 1:**
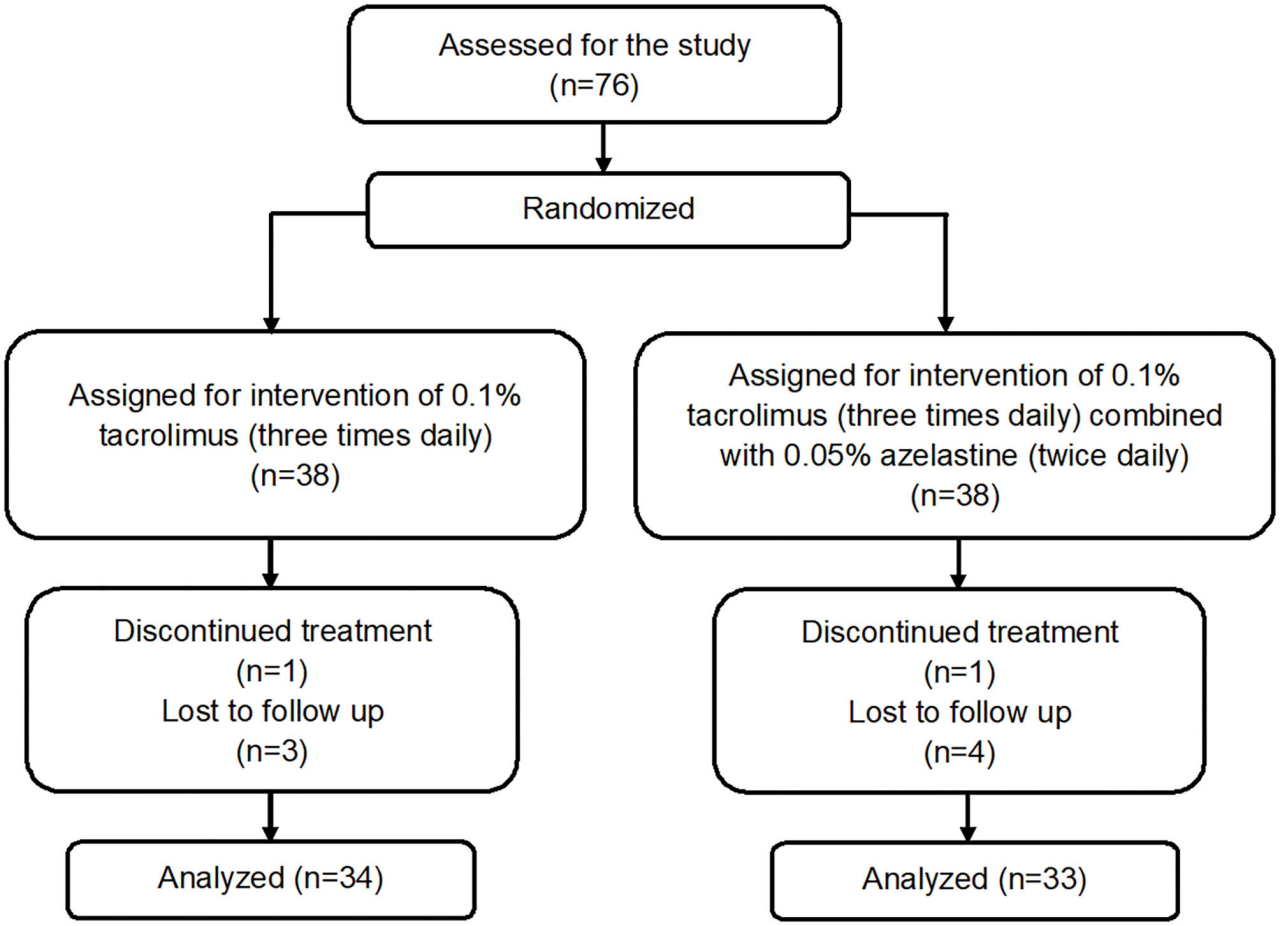
Participant flowchart.

A total of 76 patients were recruited and randomized 1:1 to the tacrolimus group and azelastine combined with tacrolimus group (Figure 1). The demographic and baseline VKC characteristics for both groups were listed in Table 1. There were no significant between-group differences in these parameters at baseline. One patient in each group discontinued therapy due to transient burning sensation upon drop instillation. Seven other patients in both groups were lost to follow up during the follow-up. There was no difference in the frequency of lost to follow-up between the two groups at 6 weeks after therapy (*p* = 0.9680), which were 8.1 and 10.8%, respectively. The remaining 67 subjects completed the treatment and were included in subsequent analyses.

**Table 1:** Patient demographic characteristics at baseline.

	**Tacrolimus group (*n* = 38 eyes)**	**Tacrolimus + azelastine group (*n* = 38 eyes)**	***p***
Age (y)	10.13 ± .2	9.72 ± .1	0.8475
Male (%)	84.2%	81.1%	0.7200
Duration of VKC (months)	23.41 ± 6.8	24.51 ± 4.2	0.5965
OSDI scores	24.99 ± .8	25.61 ± 0.9	0.8011
Conjunctival hyperemia	2.80 ± .4	2.90 ± .4	0.8131
Palpebral conjunctiva papillae	2.90 ± .5	2.80 ± .5	0.6080
Corneal involvement	2.30 ± .6	2.30 ± .6	0.9604

*VKC, vernal keratoconjunctivitis; OSDI, Ocular Surface Disease Index*.

### Changes of OSDI Scores in Both Groups

**Figure 2:**
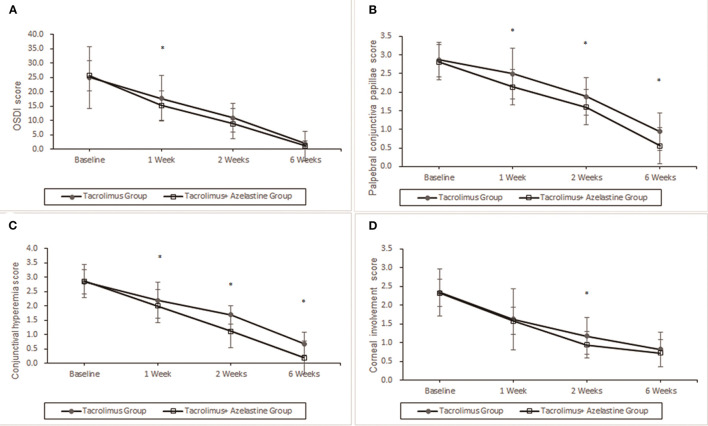
Improvement of OSDI symptoms, conjunctival hyperemia, corneal involvement and palpebral conjunctiva papillae by 0.1% topical tacrolimus eye drops alone or with 0.05% azelastine from baseline. **(A)** The OSDI score was significantly improved at 1, 2, and 6 weeks in both groups (*p* < 0.0001). Significant additive effect was observed with adjunctive topical 0.05% azelastine use at 1 week after therapy (*p* = 0.0085). **(B)** and **(C)** Patients treated with combined medicine have a significant beneficial effect compared with patients treated with tacrolimus alone at 1, 2, and 6 weeks in conjunctival hyperemia and palpebral conjunctiva papillae (all *p* < 0.02). **(D)** Insignificant between-group differences were found in corneal involvement at most visits, except for the less value in the combined group at weeks 2 (*p* = 0.0488). *p < 0.05 compared between groups (covariance analysis). OSDI, ocular surface disease index.

For the primary outcome, notable improvements from baseline in OSDI score were seen in both groups at week 1, 2, and 6 (all *p* < 0.0001). At 1 week after the initiation of therapy, the combination group showed more reduction in OSDI score from baseline (10.30 ± .9) compared with the monotherapy group (7.30 ± .7, *p* = 0.0085). However, the between-group differences in OSDI values were statistically insignificant at week 2 and week 6 (*p* = 0.1299 and *p* = 0.6073, respectively) (Figure 2).

### Changes of Signs Scores in Both Groups

**Figure 3:**
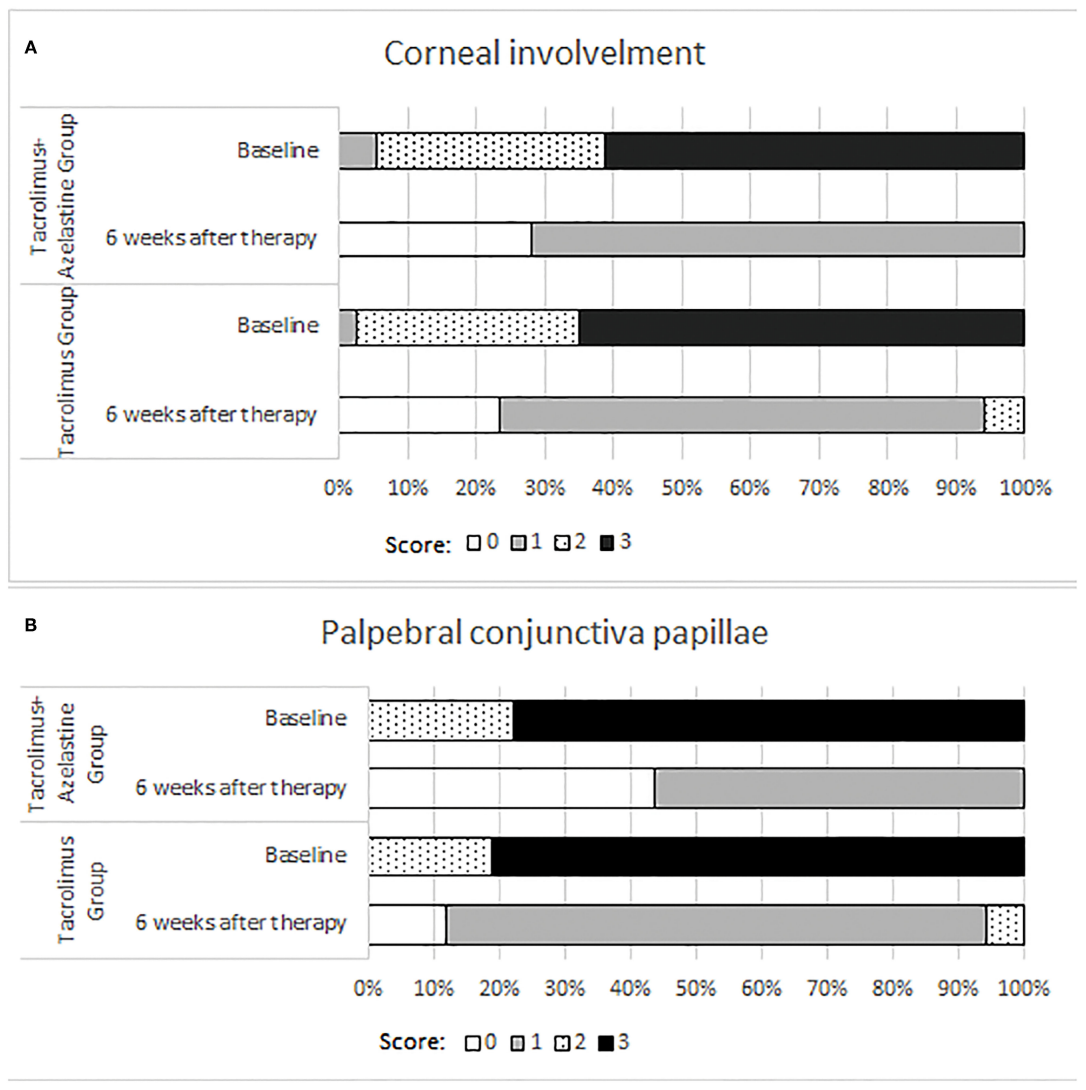
Distribution of corneal involvement **(A)** and palpebral conjunctival papillae **(B)** scores.

Significant improvements in the secondary outcomes were found at all follow-up visits. Obvious reduction in conjunctival hyperemia score was seen in both groups as early as 1 week after initiation of treatment and continued up to weeks 6 (all *p* < 0.001). At each observation after therapy, the conjunctival hyperemia score was significantly lower in the combination group than it in the monotherapy group (Table 2, Figure 2). Palpebral conjunctiva papillae score was improved with time from week 1 to weeks 6 in both groups (*p* = 0.0001 for both groups). Less scores of palpebral conjunctiva papillae were seen in the combined group than in the monotherapy group at all follow ups (Table 2, Figure 2). Significant reduction from baseline in corneal involvement score was found at all follow-up visits in both groups (all *p* < 0.0001), however, only the between-group difference at weeks 2 was showed to be significant (*p* = 0.0488) (Table 2, Figure 2). Figure 3 shows changes in distribution of severity of corneal involvement and palpebral conjunctiva papillae. Treatment in both groups yielded significantly greater improvement in corneal involvement from baseline through weeks 6. No corneal epithelial disturbance was observed in 23.5% (8/34) of patients in the monotherapy group and 27.3% (9/33) in the combined group at end of treatment. Palpebral conjunctival papillae was also significantly improved in both groups at weeks 6 after therapy. The proportions of patients with no or 1-point giant papillae were 94.1% (32/34) in the monotherapy group and 100% (33/33) in the combined group at end of treatment.

**Table 2:** Clinical signs before and after therapy.

	**Conjunctival hyperemia**			**Corneal involvement**			**Palpebral conjunctiva papillae**		
	**Tacrolimus group**	**Tacrolimus + azelastine group**	***p***	**Tacrolimus group**	**Tacrolimus + azelastine group**	***p***	**Tacrolimus group**	**Tacrolimus + azelastine group**	***p***
Baseline	2.80 ± 0.4	2.90 ± 0.4	0.8131	2.30 ± 0.6	2.30 ± 0.6	0.9604	2.90 ± 0.5	2.80 ± 0.5	00.6080
Week 1	2.20 ± 0.4	2.00 ± 0.6	0.0159	10.60 ± 0.6	10.60 ± 0.8	00.4674	20.50 ± 0.6	2.10 ± 0.7	0.0028
Weeks 2	1.70 ± 0.5	1.10 ± 0.3	<0.0001	1.20 ± 0.4	0.90 ± 0.5	0.0488	1.90 ± .3	10.60 ± 0.5	0.0064
Weeks 6	0.70 ± 0.5	0.20 ± 0.4	0.0002	0.80 ± 0.5	0.70 ± 0.5	0.3892	0.90 ± 0.4	00.60 ± 0.5	0.0021
*P*	<0.0001	0.0001		<0.0001	0.0001		0.0001	0.0001	

*P indicates the within-group differences. p indicates the between-group differences*.

### Safety

The only major adverse reaction was a transient burning sensation upon drops instillation. The incidence of adverse reactions was not significantly different between patients with (18.2%) and without (14.7%) azelastine (*p* = 0.7010). No patients experienced ocular infection during the study.

## Discussion

Repeated eye rubbing due to itching is common in VKC patients and a higher prevalence of keratoconus has been reported in those subjects (12, 13). Thus, prompt and effective alleviation of the symptoms as well as the inflammation is vital for such patients. The dual anti-histamine/mast cell stabilizing agents can take rapid onset action with immediate histamine receptor blockade, which alleviates itching and redness, along with the long-term benefit of mast cell stabilization (6). However, topical corticosteroids are usually indicated in severe cases with moderate to severe corneal involvement, numerous and inflamed limbal infiltrates or giant papillae (6). Nevertheless, they should not be considered as the first line agents due to adverse effects such as steroid-induced glaucoma and cataract (6). Topical tacrolimus has been shown to markedly improve VKC and may reduce or replace topical steroid use (6, 8, 11, 14–16). Early medical treatment with topical tacrolimus can also prevent the development of serious ocular complications of VKC, such as shield ulcers or limbal stem cell deficiency (17). In agreement with previous reports, we found that treatment with 0.1% tacrolimus eye drops alone yielded marked improvement in objective signs and OSDI score in patients with VKC after the start of therapy. Formulations 0.03 and 0.05% tacrolimus have been found to be safe and well tolerated for the treatment of severe allergic conjunctivitis (6, 18). However, the frequency of medication for low dose topical formulations is usually four times a day. In light of the burning sensation upon application, some consider 0.1% as the optimal dose since it leads to a greater symptomatic relief with similar safety profile as compared to 0.01 and 0.03% (6, 19, 20).

Since tacrolimus is effective and safe to control the signs and symptoms of VKC, the concomitant use of dual acting drugs can be questioned. Previously the combined use of tacrolimus and olopatadine in patients with VKC has been reported to have the same efficacy as the isolated use of tacrolimus (18). As for the disparity, 0.03% tacrolimus ointment was the study agent in their trial. Moreover, there was only one follow-up visit which was set at 30 days after the treatment (18). With the 0.1% tacrolimus eye drops and more intensive follow-ups, the present study highlights the benefit of tacrolimus-azelastine combination in patients with VKC which can alleviate the signs and symptoms more quickly than the isolated use of tacrolimus as early as 1 week after therapy. The combined group showed lower scores in palpebral conjunctiva papillae and conjunctival hyperemia in comparison to the monotherapy group at 1, 2, and 6 weeks after the treatment in our results, indicating better control of conjunctival disease in patients with VKC. Tacrolimus binds to FK506-binding proteins within T lymphocytes and inhibits calcineurin activity (21). Tacrolimus has also been reported to inhibit histamine release from mast cells and is thought to alleviate allergic symptoms through these mechanisms (22). Nevertheless, it was indicated tacrolimus eye drops did not have an immediate effect and required 1 to 2 weeks to be effective(11). In other words, the existing histamine in the ocular tissue may not be inhibited promptly with tacrolimus only. The major advantage of azelastine, the dual acting agent, is the rapid onset of action from immediate histamine receptor blockade, which alleviates itching and redness, along with the long-term benefit of mast cell stabilization (6). So we assume that azelastine act firstly in the 1 week before the action of tacrolimus takes place. Correspondingly, more reduction in OSDI score was observed in the combination group at week 1 after therapy. The combination of 0.05% azelastine and 0.1% tacrolimus eye drops can be the preferred therapy to achieve a fast and effective relief in pediatric patients with VKC.

Corneal involvement in VKC is quite common, including the presence of a shield ulcer, exfoliation superficial punctate keratitis and superficial keratitis. Though significant improvements from baseline were observed in both groups, the efficacy in corneal changes of the combination group was not superior to that of the monotherapy group at most visits. According to the mechanical hypothesis, the giant papillae on the upper tarsal conjunctiva cause mechanical abrasions on the cornea that may lead to shield ulcer subsequently (6, 23). Also, the chemical mediators released by eosinophils are another contributor (6, 23). This may be the reason for non-healing of shield ulcers until the inflammatory plaque is removed. A previous analysis also confirmed that the severity of the epitheliopathy was significantly associated with giant papilla and palpebral symptoms (11), indicating the remission of corneal alterations may lag behind the mitigation of palpebral conjunctiva papillae and conjunctival hyperemia. Shield ulcers have a healing time of ~1–3 months, which may explain why patients with severe VKC had a delayed reduction in the corneal epithelial sign score and an increase in disease remission (13). That would account for the insignificant difference in corneal involvement score between groups.

There are several limitations in present study, including the short time of follow-up. Visual acuity was not evaluated in this study. Because visual impairment usually occurs in VKC patients with corneal epitheliopathy, the visual prognosis and therapeutic effect of tacrolimus ophthalmic suspension for visual are crucial research themes for future studies. On the other, though we presumed the synergistic effect of tacrolimus and azelastine in anti-inflammatory, potential animal experiment is necessary. Though OSDI is usually used to reflect quality of life and also used to evaluate allergic conjunctivitis symptoms in previous studies (24, 25). Eye itching and red eye are the main symptoms of allergic conjunctivitis, which were not included in the OSDI scores. It would be better with more comprehensive assessment as the NEI-VFQ25.

In summary, the 0.1% tacrolimus eye drops alone or with 0.05% azelastine are effective and safe in treatment of VKC in children. Moreover, the combined usage brings quicker relief in conjunctival hyperemia, palpebral conjunctiva papillae and symptoms score as early as 1 week after treatment, compared with 0.1% tacrolimus alone. The beneficial effects of the combined use of tacrolimus and azelastine on signs and symptoms of VKC suggest that it may be a preferred choice in daily clinics for children.

## Data Availability Statement

The raw data supporting the conclusions of this article will be made available by the authors, without undue reservation.

## Ethics Statement

The studies involving human participants were reviewed and approved by Ethics Committee of the Eye and ENT Hospital of Fudan University. Written informed consent to participate in this study was provided by the participants' legal guardian/next of kin.

## Author Contributions

JH, JX, LG, YW, BK, and JZ conceived and designed the experiments. MC, AW, JH, JX, LG, YW, BK, and JZ performed the experiment. JX, MC, AW, and JY analyzed the data. LG, YW, BK, and JZ contributed reagents, materials and analysis tools. JX, MC, and AW wrote the paper. All authors contributed to the article and approved the submitted version.

## Funding

This work was supported by the National Natural Science Foundation of China (81970766 and 82171102), the Program for Professor of Special Appointment (Eastern Scholar) at Shanghai Institutions of Higher Learning, the Shanghai Innovation Development Program (2020-RGZN-02033), and the Shanghai Key Clinical Research Program (SHDC2020CR3052B). The sponsor or funding organization had no role in the design or conduct of this research.

## Conflict of Interest

The authors declare that the research was conducted in the absence of any commercial or financial relationships that could be construed as a potential conflict of interest.

## Publisher's Note

All claims expressed in this article are solely those of the authors and do not necessarily represent those of their affiliated organizations, or those of the publisher, the editors and the reviewers. Any product that may be evaluated in this article, or claim that may be made by its manufacturer, is not guaranteed or endorsed by the publisher.
