# Supplementary material for: Combination of 0.05% Azelastine and 0.1% Tacrolimus Eye Drops in Children With Vernal Keratoconjunctivitis: A Prospective Study
Source: Front Med (Lausanne). 2021 Sep 17;8:650083. doi: 10.3389/fmed.2021.650083 (PMC8484704; doi:10.3389/fmed.2021.650083)
Supplement: Supplementary file 1 [file Data_Sheet_1.PDF]

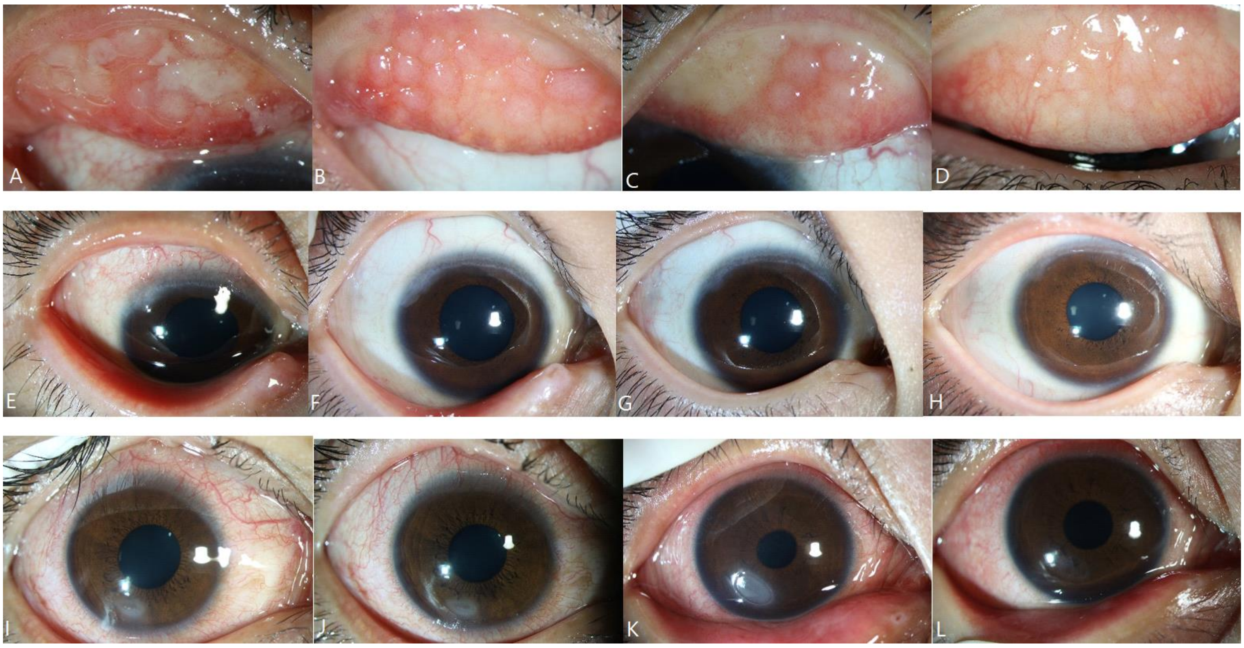

External ocular photography of signs in patients with VKC. The photos (A,B,C and D) indicate the changes of giant papillae in a VKC patient before and after treatment with 0.1% tacrolimus combined with 0.05% azelastine ophthalmic solution. Before treatment, diffuse papillary hypertrophy was observed in patient' upper tarsal conjunctivas, and some of which resembled cobblestones (A). After 0.1% tacrolimus combined with 0.05% azelastine ophthalmic solution were applied, significant shrinkage and flattening of papillae were observed at week 1, weeks 2 and weeks 6 (B, C and D). The second row comprises photos of conjunctival hyperemia in a patient with VKC before and after treatment with 0.1% tacrolimus combined with 0.05% azelastine ophthalmic solution at week 1, weeks 2 and weeks 6 (E,F, G and H). Decreased conjunctival inflammation (F, G and H) were observed after treatment. The photo in the third row exhibited persistent shield ulcer and conjunctival hyperemia (J) despite 1 week treatment with 0.1% tacrolimus compared with the photo before treatment (I). The photos (K and L) showed change of shield ulcer in the combined group. Significant

shield ulcer and conjunctival hyperemia were seen in the patient before treatment (K). After 0.1% tacrolimus combined with 0.05% azelastine ophthalmic solution were applied, decreased conjunctival hyperemia and healing of shield ulcers (L) were observed at 2 weeks after treatment.

VKC= vernal conjunctivitis.
